# The metabolic fingerprints of HCV and HBV infections studied by Nuclear Magnetic Resonance Spectroscopy

**DOI:** 10.1038/s41598-019-40028-4

**Published:** 2019-03-11

**Authors:** Gaia Meoni, Serena Lorini, Monica Monti, Francesco Madia, Giampaolo Corti, Claudio Luchinat, Anna Linda Zignego, Leonardo Tenori, Laura Gragnani

**Affiliations:** 1 University of Florence, Magnetic Resonance Center (CERM), Sesto Fiorentino, 50019 Italy; 2 Consorzio Interuniversitario Risonanze Magnetiche di Metallo Proteine (CIRMMP), Sesto Fiorentino, 50019 Italy; 3 Careggi University Hospital, Department of Experimental and Clinical Medicine, Interdepartmental Center for Systemic Manifestations of Hepatitis Viruses (MaSVE), Florence, 50134 Italy; 4 Careggi University Hospital, Infectious and Tropical Diseases Unit, Florence, 50134 Italy; 5 University of Florence, Department of Chemistry “Ugo Schiff”, Sesto Fiorentino, 50019 Italy; 6 University of Florence, Department of Experimental and Clinical Medicine, Florence, 50134 Italy

## Abstract

Few studies are available on metabolic changes in liver injuries and this is the first metabolomic study evaluating a group of HCV-positive patients, before and after viral eradication via DAA IFN-free regimens, using ^1^H-NMR to characterize and compare their serum fingerprints to naïve HBV-patients and healthy donors. The investigation clearly shows differences in the metabolomic profile of HCV patients before and after effective DAA treatment. Significant changes in metabolites levels in patients undergoing therapy suggest alterations in several metabolic pathways. It has been shown that ^1^H-NMR fingerprinting approach is an optimal technique in predicting the specific infection and the healthy status of studied subjects (Monte-Carlo cross validated accuracies: 86% in the HCV vs HBV model, 98.7% in the HCV vs HC model). Metabolite data collected support the hypothesis that the HCV virus induces glycolysis over oxidative phosphorylation in a similar manner to the Warburg effect in cancer, moreover our results have demonstrated a different action of the two viruses on cellular metabolism, corroborating the hypothesis that the metabolic perturbation on patients could be attributed to a direct role in viral infection. This metabolomic study has revealed some alteration in metabolites for the first time (2-oxoglutarate and 3-hydroxybutrate) concerning the HCV-infection model that could explain several extrahepatic manifestations associated with such an infection.

## Introduction

Hepatitis C virus (HCV) global prevalence is estimated to be 2% (180 million people), therefore the number of people at risk of developing HCV-related chronic liver disease is substantial. In fact, HCV-chronic infection causes progressive liver damage that it is usually assessed through different methods, and, one of the most common is the METAVIR score, that is useful in evaluating the stage of fibrosis, ranging from the milder grade F0 (no fibrosis) to F4 (cirrhosis), via the histopathological evaluation of patients with hepatitis C in a liver biopsy^1^. Cirrhosis is the end-stage of every chronic liver disease, and is a major risk factor for the development of hepatocellular carcinoma HCC.

Anti-HCV therapy has been based on the administration of Pegylated-Interferon (Peg-IFN) plus Ribavirin (RBV) for almost 15 years; the treatment of the HCV-infection has recently experienced a remarkable advancement with the introduction of new direct-acting antivirals (DAAs). In large real-life cohorts DAAs allow Sustained Virological Response (SVR) rates exceeding 90%, 95–97% in compensated cirrhosis and 85–90% in subjects with more advanced liver disease^2–4^.

In the near future, DAA regimens will probably reduce the incidence of hepatic decompensation and the development of HCC^5^.

A significant contribution to this new approach has been provided by the so called “-omic” techniques over the last fifteen years. This term refers to innovative technology platforms such as genetics, genomics, proteomics, and metabolomics, which allow us to detect and identify many different molecules that are present in the body. A number of diagnostic and prognostic molecular markers were previously identified by -omics analyses of HCV and HBV infections, but usually, these studies were focused on specific aspects/steps of the disease natural history. Most of the literature about systematic -omics studies made in the scales of genomics, transcriptomics and proteomics in the HCV and HCV field are, in fact, related to HCC susceptibility, which is a priority topic for research^6,7^. Other studies comparatively analyzed different stages of chronic liver damage in HCV and HBV infections, always with the aim to find early cancer biomarkers using proteomics^8,9^.

Metabolomics is the latest of the “-omic” techniques and has emerged as an exciting tool for biomedical researchers^10^. Metabolomics is the study of the metabolome, i.e. the whole ensemble of small molecules (metabolites) present in body fluids, cells or tissues^11^. The chemical nature and the relative abundance of detectable metabolites can be viewed as the fingerprint that characterizes the physio-pathological state of an individual^12^. While genomics tells us “what could happen”, metabolomics tells us “what is happening”. Thus, metabolomics plays a significant role in bridging the phenotype-genotype gap and assists us not only in linking gene to function but also in gaining an entirely new insight into how organisms respond to exogenous stimuli and how individuals evolve over time^13,14^. Proton nuclear magnetic resonance (^1^H-NMR) spectroscopy is one of the main analytical tools that is used in metabolomics. It allows simultaneous identification and quantification of hundreds of small molecules, creating a disease-specific metabolomic profile/fingerprint^15^.

Presently, there are few studies concerning HCV-infection that are based on a metabolomic approach^16–19^; however, the majority of metabolomic analyses were performed on subjects with chronic liver damage with no stratification according to etiology^20,21^. The most interesting data regarding HCV infection and antiviral therapy effects are reported by Saito *et al*. by comparing the metabolic profiles of 10 patients with chronic HCV infection who failed treatment with Peg-IFN and RBV, in those 10 subjects, who obtained an SVR after the same therapy. Despite the limited population and the absence of appropriate controls, this study suggests that it could be interesting to evaluate the HCV infection pattern with a metabolomics approach on a wider and better characterized population. The need of a well-designed metabolomics analysis on large cohorts of well-defined patients undergoing standardized therapies, evaluating samples drawn at different time points corresponding to specific clinical situations (before treatment, after treatment at the time of judgement of efficacy, and subsequently at interesting end-points) was also suggested by the most comprehensive review on this topic^22^.

The new direct antiviral therapies, which have a direct target on the replication of the virus, provide an ideal model of study: indeed, the lack of possible confounding effects caused by biologically active molecules with a systemic action such as interferon should allow us to obtain a real metabolic picture of the patient.

The aim of this study was to evaluate a group of HCV-positive patients, before and after viral eradication through DAA IFN-free regimens, through an NMR-based metabolomic approach, comparing their fingerprints to naïve hepatitis B virus (HBV) positive patients and healthy controls (HC).

## Results

NMR spectra of 294 serum samples were acquired respectively from 67 HCV positive patients collected at three different time points corresponding to baseline (during the infection), 12 weeks and 24 weeks after the sustained virological response (namely SVR12 and svr24), 50 naïve HBV infected patients and 43 serum samples from healthy controls (HC).

As a preliminary unsupervised approach, principal component analysis (PCA) was used to obtain a simplified view of the variation in the data, and to check the sample quality thereby excluding the presence of outliers.

### DAA induced metabolomic changes in HCV patients

**Figure 1:**
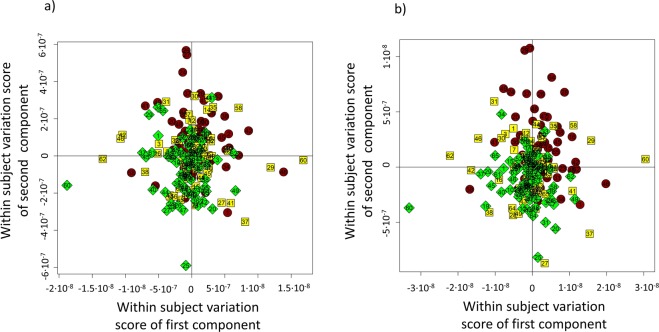
Score plots of MPLS pairwise discrimination of HCV infected patients at baseline (red dots), and post treatment subjects respectively SVR12 (yellow squares) and SVR24 (green rhombus); (**a**) model built using bucketed 1D NOESY spectra; (**b**) model built using bucketed CPMG spectra. Given the paired approach of the analysis, each number reported on the symbols, refers to the serum of a patient sampled at the three different time points.

With the aim of analyzing within-subject metabolic changes introduced by DAAs therapy, multilevel partial least-squares analysis (MPLS) was performed on 67 × 3 HCV serum samples collected at three different times (baseline-HCV, 12 weeks- and 24 weeks-after SVR). Multilevel PLS models were built on 1D NOESY and CPMG spectra (Fig. 1) and both proved satisfactory in discriminating baseline from SVR12 and SVR24 subjects (64.5% predictive accuracy from the 1D NOESY model and 70% from the CPMG model).

As it emerges from the confusion matrices (Table 1), SVR12 samples mingle with SVR24 samples, indicating that, at the metabolic level, the treatment effect is already visible 12 weeks after therapy and patient profiles remain stable for at least the next twelve weeks of treatment.

**Table 1:** MPLS analysis on 1D NOESY and CPMG spectra.

Confusion matrix of NOESY spectra				Confusion matrix of CPMG spectra			
	*HCV*	*SVR12*	*SVR24*		*HCV*	*SVR12*	*SVR24*
*HCV*	**79**.**7**	12.3	8	*HCV*	**82**.**1**	11	6.9
*SVR12*	10.1	**54**.**6**	35.3	*SVR12*	11	**61**.**6**	27.4
*SVR24*	8.1	32.6	**59**.**3**	*SVR24*	5.1	27.4	**67**.**4**
Overall predictive accuracy			**64**.**5%**	Overall predictive accuracy			**70%**

Confusion matrix and prediction accuracy of Monte Carlo cross-validation are reported for both models.

We tested the influence of therapy duration (12 or 24 weeks), administration of ribavirin (RBV), HCV genotypes and METAVIR score on the metabolic fingerprint.

Concerning treatment duration and RBV administration, neither multivariate analysis on spectral buckets (predictive accuracy <50%) nor univariate analysis on metabolite levels highlighted significant differences among HCV subgroups.

Regarding the analysis on HCV genotypes, considering the small number of individuals *per* group as the main limitation, and thus the impossibility of comparing all of them accurately, a comparison between the most common HCV viral genotypes (Gt1a/1b and Gt 2a/2c) was attempted without obtaining a reliable discrimination (predictive accuracy = 61%).

On the other hand, the analysis of HCV subgroups stratified on METAVIR score revealed interesting differences.

**Figure 2:**
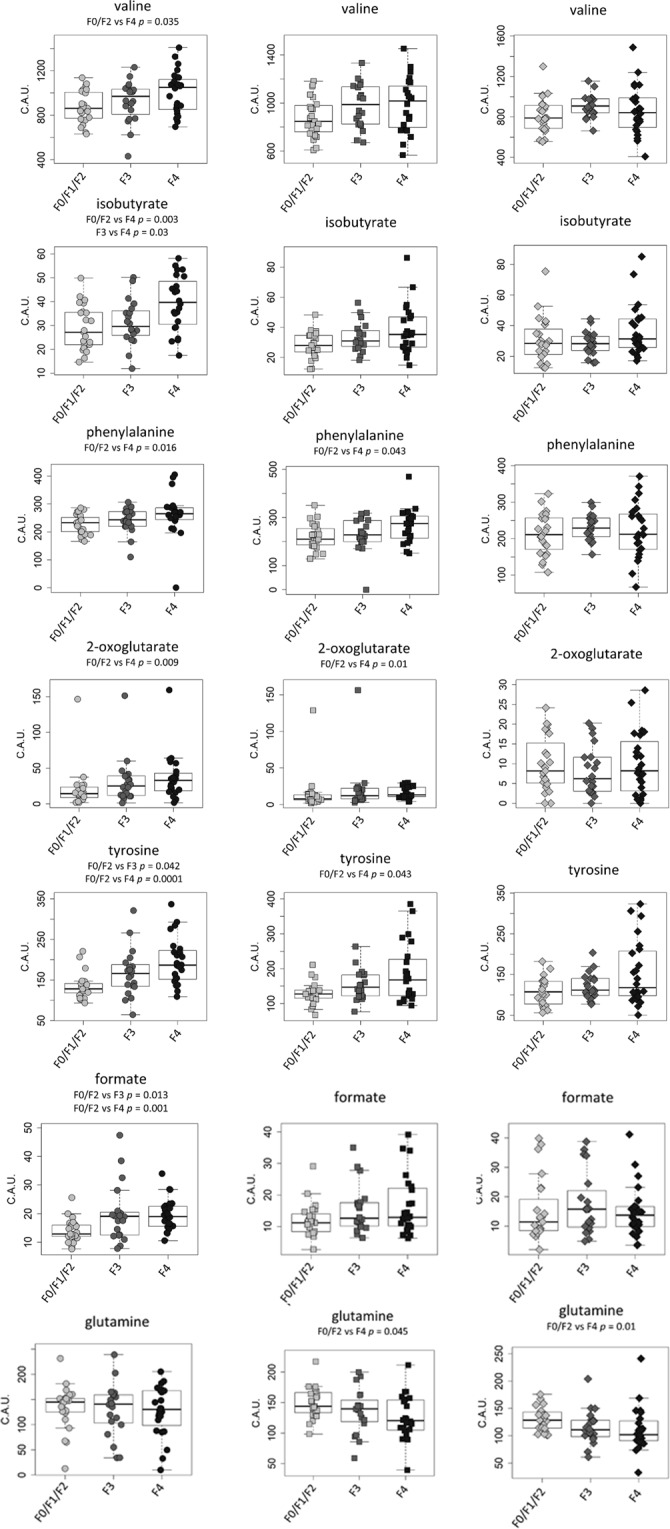
Concentration levels of metabolites in advanced and mild fibrosis. Box plots of the metabolites differentially concentrated (concentration expressed in arbitrary units, C.A.U) in F0/F1/F2 (light grey, n = 23), F3 (dark grey, n = 20) and F4 (black, n = 24) for each time point: baseline (circles), SVR12 (squares) and SVR24 (rhombus). For each comparison, the adjusted *P*-value (Kruskal-Wallis test followed by Dunn post-hoc) is also reported.

The NMR spectra of serum samples from HCV patients with a high score of fibrosis (F3 and F4) and without fibrosis or lower fibrosis (F0/F1 and F2) were analyzed at each time point (Baseline, SVR12, and SVR24) using multivariate and univariate statistical analyses. The OPLS-DA (Orthogonal Projection to Latent Structures – Discriminant Analysis) test was not effective in discriminating advanced fibrosis from mild fibrosis, but the concentration levels of several metabolites were significantly altered, as assessed by univariate tests (Fig. 2, Supplementary Tables 4–6).

In baseline samples, valine, isobutyrate, phenylalanine, 2-oxoglutarate, tyrosine, and formate significantly decreased from F4 to F0/F1, and glutamine only increased from F4 to F0/F1.

### Comparison among naïve HCV, naïve HBV and healthy subjects

We also performed comparative analyses among the groups and the 67 HCV baseline samples. The 50 naïve HBV patient samples and the 43 serum samples from healthy controls were used to build two OPLS-DA models, one on NOESY spectra and the other on CPMG spectra, with the aim of defining a statistical model able to accurately predict the healthy or pathological state of a certain subject, simply using a single serum NMR spectrum.

**Figure 3:**
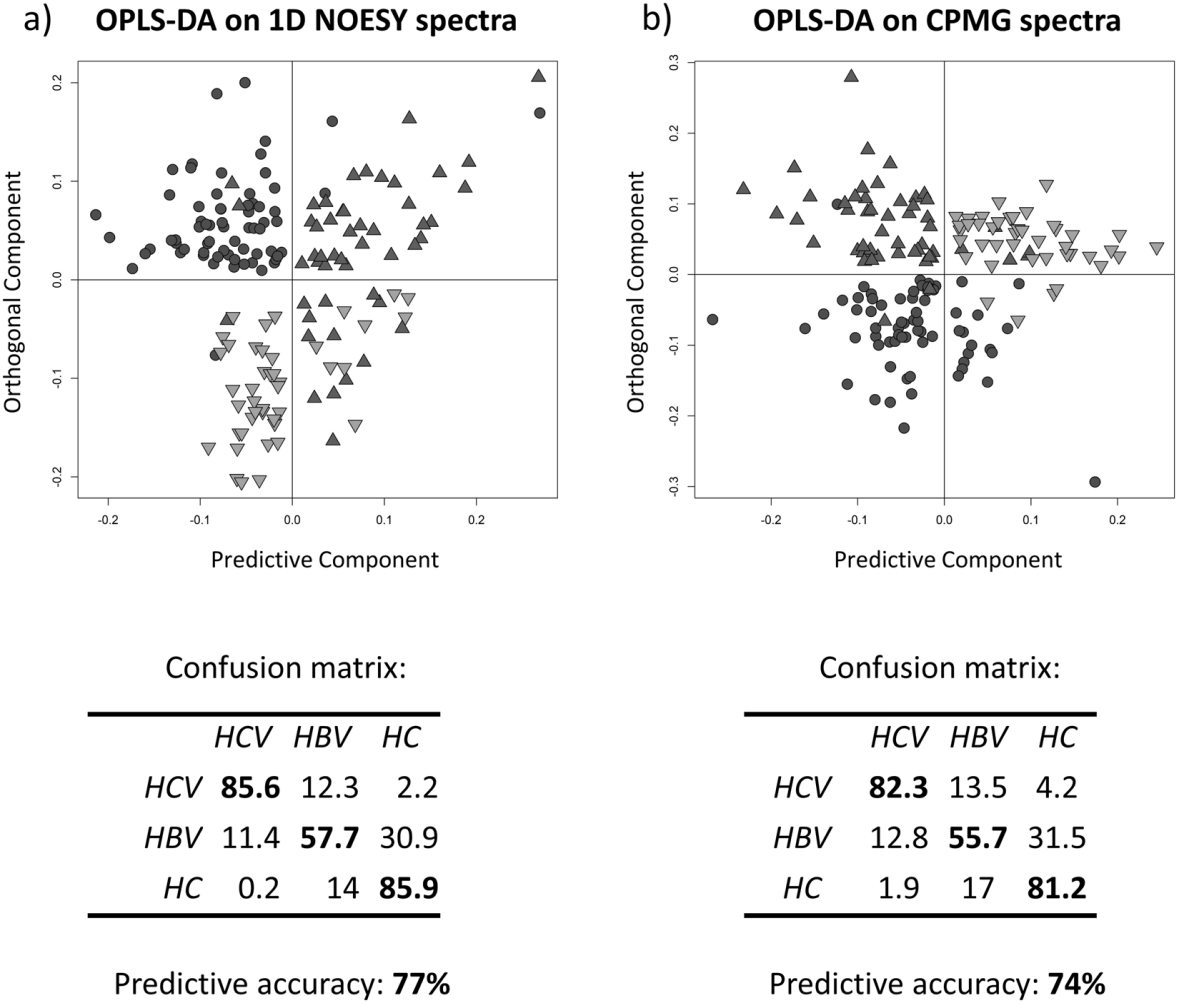
OPLS-DA on HCV, HBV and HC ^1^H-NMR serum spectra. Score plots of OPLS-DA, discriminating HCV (dots, n = 67), HBV patients (triangles, n = 50) and HC (inverted triangles, n = 43); (**a**) NOESY experiment; (**b**) CPMG experiment. Confusion matrix and prediction accuracy of Monte Carlo cross-validation are also reported for both models.

Both OPLS-DA models show a clear-cut discrimination between HCV, HBV and HC, with an overall cross-validated accuracy of 77% on NOESY and 74% CPMG models respectively (Fig. 3a,b). For the sake of completeness, confusion matrices and OPLS-DA score plots of each comparisons are available in Supplementary Materials (Supplementary Fig. 1 and Table 1), with an overall cross-validated accuracy of 86% (using NOESY experiments to build the model) and 84% (using CPMG experiments to build the model) by comparing HCV vs HBV serum profile, 98.7% (NOESY) and 98% (CPMG) by comparing HCV vs HC, and an overall cross-validated accuracy of 80% (NOESY) and 77.6% (CPMG) by comparing HBV subjects with healthy controls (HC).

### Detailed analysis of metabolites

With the aim of identifying metabolite level variations related to changes introduced by the therapy, pairwise univariate analysis was applied on thirty-one assigned metabolites.

When the post-hoc Nemenyi test was applied on baseline (HCV), SVR12 and SVR24 serum samples, significant differences were evident (Table 2): 2-oxoglutarate levels progressively decrease from baseline (HCV) to SVR12 and SVR24; 3-hydroxybutyrate, formate and acetate levels are significantly higher in serum samples before therapy; histidine, ornithine, phenylalanine, creatine and tyrosine levels are lower in SVR24 if compared with HCV and SVR12. These alterations are informative both to indicate the presence of HCV/pathological state biomarkers in infected serum samples (e.g. 2-oxoglutarate, 3-hydroxybutyrate, acetate, and formate), and for the effect of the therapy on patients’ metabolism. A simplified list of the most contributing metabolic pathways is reported in Table 3.

**Table 2:** Pairwise metabolites analysis of baseline (HCV), SVR12 and SVR24.

Metabolites	*P*-value (effect size)		
	Groups	*HCV*	*SVR12*
*2-oxoglutarate*	*SVR12*	0.0004**↑ (m)**	—
	*SVR24*	0.00004**↑(m)**	0.041**↑(s)**
*3-hydroxybutyrate*	*SVR12*	0.03**↑(s)**	—
	*SVR24*	0.03**↑(s)**	0.608(n)
*acetate*	*SVR12*	0.005**↑(s)**	—
	*SVR24*	0.02**↑(s)**	0.095(s) ↑
*creatine*	*SVR12*	0.663(n)	—
	*SVR24*	0.015**↑(s)**	0.025**↑(s)**
*formate*	*SVR12*	0.03**↑(s)**	—
	*SVR24*	0.0071**↑(s)**	0.996(n)
*histidine*	*SVR12*	0.769(n)	—
	*SVR24*	0.004**↑(s)**	0.033**↑(s)**
*ornithine*	*SVR12*	0.88(n)	—
	*SVR24*	0.001**↑(s)**	0.98(n)
*phenylalanine*	*SVR12*	0.306(n)	—
	*SVR24*	0.0002**↑(s)**	0.27**(n)**
*tyrosine*	*SVR12*	0.22(s)	—
	*SVR24*	0.00001**↑(s)**	0.02**↑(s)**
*lactate*	*SVR12*	0.42(n)	—
	*SVR24*	0.04**↑(s)**	0.14(n)

Adjusted *P*-values (FDR) are reported for each comparison. ↑ in baseline (HCV) vs SVR12 and SVR24, means higher metabolite levels in HCV; ↑ in SVR12 vs SVR24 means higher levels in SVR12. Values in bold are significantly altered (*P*-value < 0.05), effect size is also reported for each comparison (n = negligible, s = small, m = medium, l = large).

**Table 3:** Pathways analysis.

Pathway Name	Metabolites	*P-*value	Holm P-value	FDR	Impact
Lysine biosynthesis	2-oxoglutarate	9.4 × 10^−7^	4.4 × 10^−5^	4.4 × 10^−5^	0
Nitrogen metabolism	phenylalanine; tyrosine; histidine; formate	1.5 × 10^−5^	9.2 × 10^−4^	9.2 × 10^−4^	0
Ubiquinone and other terpenoid-quinone biosynthesis	tyrosine	1.6 × 10^−4^	0.01	0.004	0
Pyruvate metabolism	lactate; formate; acetate; pyruvate	2.4 × 10^−4^	0.02	0.01	0.24
Phenylalanine, tyrosine and tryptophan biosynthesis	phenylalanine; tyrosine	3.9 × 10^−4^	0.02	0.01	0.01
Glycine, serine and threonine metabolism	creatine	0.002	0.07	0.02	0.24
Aminoacyl-tRNA biosynthesis	phenylalanine; tyrosine	0.003	0.12	0.02	0.06
Selenoamino acid metabolism	acetate	0.004	0.14	0.02	0.003
Taurine and hypotaurine metabolism	pyruvate; acetate	0.004	0.163	0.02	0.05
Alanine, aspartate and glutamate metabolism	pyruvate; 2-oxoglutarate	0.004	0.17	0.028	0.44
D-Glutamine and D-glutamate metabolism	2-oxoglutarate	0.005	0.19	0.02	0.14
Histidine metabolism	2-oxoglutarate; histidine	0.005	0.19	0.021	5.1 × 10^−4^
Cysteine and methionine metabolism	pyruvate	0.005	0.2	0.02	0.05

An integrated analysis based on MetaboAnalyst 4.0 software built on significantly altered metabolites in patients undergoing therapy: view of most contributing pathways; *P-*value is the original p value calculated from the enrichment analysis; Holm *P-*value is the *P-*value adjusted by Holm-Bonferroni method; the FDR is the *P-*value adjusted using False Discovery Rate; Impact is the pathway impact value calculated from pathway topology analysis.

**Figure 4:**
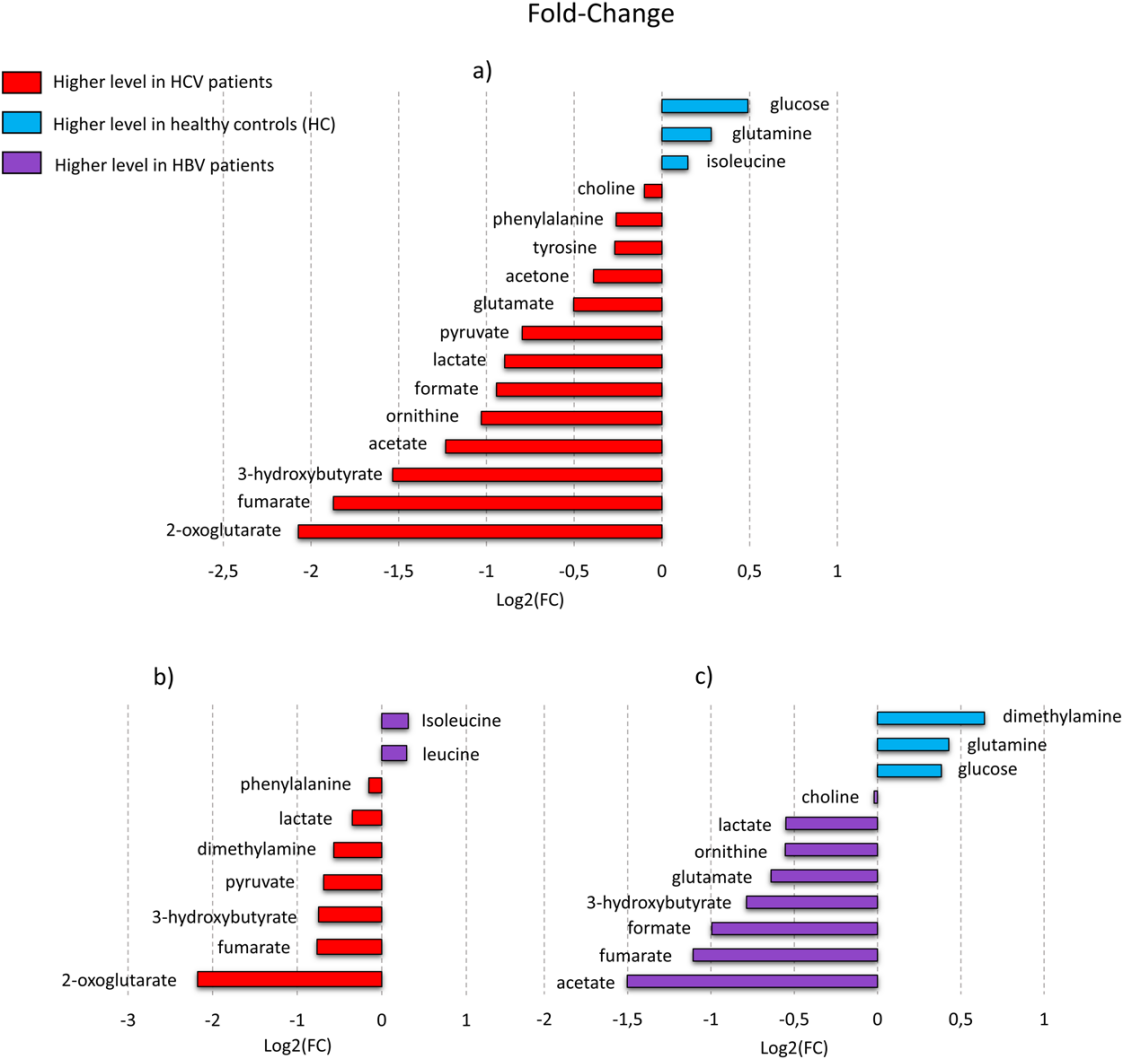
Boxplots of Fold-Change values (FC) for the significantly altered metabolites (*P*-value < 0.05) in HCV, HBV and HC serum samples. (**a**) Comparison between HCV (n = 67) and healthy subjects (HC, n = 43), negative FC values mean higher metabolite levels in HCV serum samples; (**b**) comparison between HCV and HBV patients (n = 50), positive FC values mean higher metabolite levels in HBV sera; (**c**) comparison between HBV and HC subjects, negative FC values mean higher metabolite levels in HBV serum samples.

Afterwards, with the aim of characterizing the direct action of HCV in metabolic pathways, serum levels of each metabolite in patients infected by HCV and HBV were compared between themselves (Fig. 4b) and each of them with healthy controls (HC) as shown in Fig. 4a,c (Supplementary Table 2). For each of those comparisons (HCV vs HC, HBV vs HCV, HBV vs HC) using statistically significant altered metabolites, pathways analysis was performed and is reported in Supplementary Fig. 2 and Table 3.

## Discussion

To the best of our knowledge, the present NMR metabolomics-based study is the first that examines the HCV serum profile of infected patients before and after viral clearance by DAAs, and the first one comparing a high number of naïve HCV patients to naïve HBV patients and to healthy controls.

NMR metabolomic approach has been widely proven to be a fast and reliable technique to generate serum metabolite profiles of groups under study by using intact biospecimens with minimal samples preparation^15^.

This investigation clearly shows differences in the metabolomic profile of HCV patients before and after effective DAA treatment. Indeed, the multilevel PLS (MPLS) model built on bucketed ^1^H-NMR serum CPMG spectra has proven to be effective in characterizing the serum metabolomic fingerprint of patients undergoing DAAs therapy (overall predictive accuracy of 70%). The use of a selective CPMG pulse is commonly used in NMR metabolomic studies to gain a better resolution of low molecular weight molecules (metabolites) with respect tothe standard sequence such as 1D NOESY (in which small molecules are penalized by the intense and wide signals arising from macromolecules such as lipids, lipoproteins and albumin) as shown in Supplementary Fig. 3 where 1 H CPMG and NOESY spectra are reported to better explicate these differences. In this case, the slightly better accuracy gained by CPMG model could be used to speculate a major contribution of low molecular weight molecules in characterizing the within-subject changes introduced in the individual metabolic profile through DAAs therapy.

This result suggests a metabolic shift after therapy and viral eradication, which can be recorded through ^1^H-NMR spectroscopy of intact serum samples. Significant changes in metabolite levels in patients undergoing therapy suggest alterations in several pathways such as lysine biosynthesis (2-oxoglutarate), nitrogen metabolism (phenylalanine, tyrosine, histidine, formate), ubiquinone and other terpenoid-quinone biosynthesis (tyrosine), pyruvate metabolism (lactate, formate, acetate, pyruvate), phenylalanine, tyrosine and tryptophan biosynthesis (phenylalanine, tyrosine), glycine, serine and threonine metabolism (creatine), aminoacyl-tRNA biosynthesis (phenylalanine, tyrosine), selenoamino acid metabolism (acetate), taurine and hypotaurine metabolism (pyruvate, acetate), alanine, aspartate and glutamate metabolism (pyruvate, 2-oxoglutarate), D-Glutamine and D-glutamate metabolism (2-oxoglutarate), histidine metabolism (2-oxoglutarate, histidine) and cysteine and methionine metabolism (pyruvate). Some of these pathways were already identified using integrative multiomics analyses (whole genome and transcriptome sequencing) in the context of the identification of prognostic biomarkers in hepatitis B virus-related hepatocellular carcinoma^23^. Other studies investigated HCV or HBV infection and response to therapy using micro RNAs networks^24^, transcriptomics^25^, proteomics^26–28^. However, a direct comparison with the present results is difficult due the different study designs and the different clinical settings.

Multivariate analysis on spectral buckets (predictive accuracy < 50%) and univariate analysis on metabolite levels, did not highlight post-therapy significant differences among HCV subgroups stratified according to treatment duration and RBV administration. Unfortunately, we could not perform a reliable statistical analysis regarding the different viral genotypes since the genotypes were not equally represented. In fact, from a metabolic point of view, genotype 3 seems to be the most interesting one, being associated with severe steatosis, but also with an accelerated fibrosis progression rate and increased oncogenesis^29^. Even if no significantly altered metabolite levels were detected between the two more numerous genotype subgroups (genotype 1b and 2), a further analysis to evaluate genotype 3 contribution on metabolic fingerprints, with special focus on lipid metabolism, could be useful to clarify the genotype-related differences in liver disease progression.

The OPLS-DA analysis of naïve HCV patient sera stratified according to METAVIR score and grouped in F0-F2, F3, and F4 did not allow us to discriminate between advanced and mild fibrosis. Conversely, Cano *et al*. proposed a diagnostic algorithm built using two sphingomyelins and two phosphatidylcholines measured in sera by mass spectrometry (MS), which is capable of accurately classifying rapid and slow fibroses after liver transplantation^30^.

Nevertheless, in the present study, differences in a few metabolite levels among the three METAVIR subgroups were significant, suggesting that different concentrations of metabolites characterize different fibrosis scores. In accordance with other authors^30,31^, tyrosine and formate increased ranging from no/mild fibrosis to severe fibrosis, and their role as potential fibrosis severity biomarkers should be investigated in depth.

In addition, the differences in metabolite levels between higher and lower fibrosis scores, detected in patients before therapy, decreased after SVR, confirming that altered metabolites are restored, most likely due to liver damage regression induced by viral eradication.

The two OPLS-DA models showed a clear-cut discrimination between baseline HCV, naïve HBV and HC, with an overall cross-validated accuracy of 77% on NOESY and 74% CPMG models respectively. While the main metabolic differences recorded among the three metachronous HCV-samples, collected from the same patient, are due to low weight molecules (CPMG analysis), the comparison between HCV and HBV positive sera showed a better discrimination in the NOESY spectra, which also contain signals arising from high molecular weight molecules (i.e. those involved in lipid metabolism) as shown in Supplementary Fig. 3. The contribution of the lipids related peaks to the separation is confirmed by the loading plot of the NOESY OPLS-DA model (Supplementary Fig. 4). This information leads to two different speculations; i) In HCV patients, antiviral therapy is able to positively change the metabolic pattern of small molecules but, in the short post-therapy follow-up it does not seem to affect the serum pattern of molecules involved in lipid metabolism. This is in agreement with steatosis maintenance after SVR in HCV patients, except for those infected with Gt3 (viral genotype 3), which is often observed in clinical practice^32^; indeed, HCV Gt 3 is more likely to cause steatosis than the other genotypes^33^, but Gt3-related steatosis is significantly reduced by SVR while there was no change in steatosis, after SVR, among patients infected with the other viral genotypes^32^. As already mentioned, in our analysis, the number of Gt3 infected patients was too low to allow statistical analysis, but our data, supported by clinical observations, suggest that the other genotypes are able to induce a metabolic perturbation in lipid metabolism that persists after viral eradication; ii) it is well-known that while HBV infection is generally free from insulin resistance, steatosis and increased cardiovascular risk, HCV is associated with dysmetabolic syndrome, featuring steatosis, hypocholesterolemia and insulin resistance, determining a substantially increased cardiovascular risk^34^. Our results support these findings, showing a completely different action of the two major hepatitis viruses on basal metabolism, and the contribution of liver damage severity on the perturbation of metabolic pathways seems to have a lesser impact than the kind of virus.

In addition, an analysis of specific metabolite levels was performed. 2-oxoglutarate levels progressively decrease from baseline HCV infected patients towards SVR12 and SVR24; 3-hydroxybutyrate, formate and acetate levels were significantly higher before therapy; histidine, ornithine, phenylalanine, creatine, and tyrosine levels are lower in SVR24 if compared to the baseline and SVR12.

The OPLS-DA model built on NOESY spectra of HCV, HBV infected patients and healthy controls proved to be effective in predicting the specific infection and the healthy status of studied subjects (predictive accuracies: 85.6% HCV, 57.7 HBV and 85.9% HC). Moreover, analysis on metabolite levels among these three groups reveals significantly higher levels (adjusted *P*-values < 0.05) of 2-oxoglutarate, fumarate, 3-hydroxybutyrate, acetate, ornithine, formate, lactate, pyruvate, glutamate, acetone, tyrosine, phenylalanine, and choline in HCV infected patients and lower levels of glucose, glutamine and isoleucine if compared to HC; higher levels of 2-oxoglutarate, fumarate, 3-hydroxybutyrate, pyruvate, dimethylamine, lactate and phenylalanine and lower levels of isoleucine and leucine if compared to HBV infected patients.

Among these metabolites, there are molecules that were found to be upregulated in a previous *in vitro* study using primary human hepatocytes infected with HCV^35^; in fact, Levy and coworkers observed a HCV-induced upregulation of glycolysis and ketogenesis, which are completely confirmed by the metabolic signature of HCV treatment-naïve patients emerging from our analysis. The increased levels of pyruvate, lactate, acetate, and 3-hydroxybutyrate seem to support the hypothesis that the virus induces glycolysis over oxidative phosphorylation in a similar manner to the Warburg effect in cancer^36^. As the authors speculated that the global upregulation of metabolic pathways was a virus-induced effect rather than the host response to infection, our results seem to confirm this hypothesis^35^. Indeed, we compared HBV- and HCV-infected patients and we noted a different behavior of several metabolites in the two sets of patients, suggesting once again that the perturbation could be attributable to a direct action of HCV rather than to the host response, which is similar in the two infections.

Our results apparently differ from those reported by Saito and colleagues^19^ that compared, through capillary electrophoresis and liquid chromatography–mass spectrometry, the metabolic profiles of 10 HCV-patients who failed treatment with Peg-IFN and RBV, to 10 subjects, who obtained an SVR after the same therapy; PegIFN/RBV therapy was shown to reduce the level of oxidative stress in patients with chronic HCV infection. On the other hand, we obtained results consistent with the theory that in HCV infection the glycolysis toward lactate production is increased in aerobic conditions^35^. The increase of oxidative stress (Reactive Oxygen Species production) leads the cells towards the glycolytic way with lactate production^37^ and the two effects seem to be strongly linked and coexist^38^.

Therefore, the post-eradication lactate decrease we reported, and the reduction of oxidative stress observed by Saito and colleagues, could represent “two sides of the same coin”, as the effects of metabolic changes induced by HCV that revert towards normality once viral eradication is obtained, independently from the kind of therapy.

Especially interesting are the high levels of 2-oxoglutarate and 3-hydroxybutyrate in HCV baseline samples compared to HC and HBV samples. In fact, both metabolites were found to be altered in association to cardiovascular diseases and were therefore previously identified as potential biomarkers of cardiovascular risk. In more detail, high levels of 2-oxoglutarate were present in patients with heart failure^39,40^, with chronic resistant hypertension^41^ and could more generally be considered as biomarkers of cardiovascular risk^42^. In addition, high levels of the ketone-body 3-hydroxybutyrate were also found to be associated with cardiovascular diseases^43,44^ and were identified as part of a panel of cardiovascular risk biomarkers through different analyses^45,46^. 3-hydroxybutyrate was also identified as one of the first ketone-bodies to increase in the diabetic ketoacidosis and defined as diabetes biomarkers (even if an early predictive biomarker) in humans and in *in vivo* murine models^47–50^.

Interestingly, cardiovascular diseases and diabetes mellitus are two of the several extrahepatic manifestations associated with chronic HCV – but not HBV - infection^51–54^. Therefore, it could be speculated that HCV has a direct role in altering biochemical pathways of basal metabolism, and this could aid in explaining the association with some of the most frequent extrahepatic manifestations such as cardiovascular diseases and diabetes mellitus. In order to ascertain this hypothesis, further, dedicated studies using *in vitro* models of perturbations of biochemical pathways should be performed; in addition, longer post-therapy follow-up studies evaluating metabolomic fingerprints of HCV SVR patients could confirm that the restoration of increased metabolites, such as 2-oxoglutarate and 3-hydroxybutyrate, is related to the decreased mortality rate due to extrahepatic causes which is reported in large cohort studies^22,55,56^.

Our study provided an interesting picture of metabolic fingerprints of HCV infection compared to HC and, mostly, to HBV-infected patients, suggesting that the two viruses have a different impact on cellular metabolism; metabolomic approach is able to show alterations in several metabolic pathways that, concerning HCV-infection model, could explain some of the several extrahepatic manifestations associated with such an infection. Of course, the interesting results we reported require a functional confirmation through further dedicated studies and bigger multicentrical cohorts of patients.

Our results show how metabolomics can lead to the design of new rapid, economic, and sensitive diagnostic tests, which are also useful for prognostic purposes.

In particular, a metabolomic approach has proved to be an effective tool to predict variations in response to treatment. Collecting multiple samples for each individual at different time points represents potential for such studies, enabling us to eliminate inter-individual variations and constructing a more accurate picture of the treatment effect on the population studied. Longer post-therapy follow-up epidemiological studies evaluating metabolomic fingerprints of HCV SVR patients could confirm that the restoration of altered metabolite levels is related to the decreased mortality rate due to extrahepatic causes.

## Patients and Methods

### Patients and serum collection

Sera were collected before therapy (baseline), at SVR12, and SVR24 time-points, from 67 HCV patients (age 63.4 ± 1.22) successfully treated with IFN-free DAA regimens; as controls, we tested serum samples from 50 naive subjects with chronic HBV infection (pathological controls; HBV, age 56.6 ± 2.3) and 43 healthy donors (healthy controls; HC, age 50.7 ± 18.9, 58% females). HCV-positive patients were recruited from the outpatient clinic of the Center for Systemic Manifestations of Hepatitis Viruses (MaSVE), University of Florence, Italy, while HBV subjects and HCs were recruited from the MaSVE Center and from the Infectious and Tropical Diseases Unit of the University of Florence, Florence, Italy.

Blood was drawn, in the absence of an anticoagulant, following routine phlebotomy methods and immediately after centrifugation the serum was placed at −80 °C to be subsequently examined by NMR.

The main baseline demographic and virological characteristics of the HCV and HBV samples were stratified in the three groups, as previously described (REF), and are reported in Table 4. No demographic information was available for HCs who were negative for HCV and HBV as well as other viral infections and patients were not known to suffer from any significant illnesses relevant to the present study.

**Table 4:** Main demographic, hepatovirological, and clinical baseline characteristics of HCV and HBV infected patients before treatment.

	HCV	HBV	*P-value*
	(n = 67)	(n = 50)	
Age (years)*	63.4 (±1.22)	56.6 (±2.3)	≤0.0159
Gender (male/female)	26/41	31/19	<0.015
**Metavir score^:**			
F0-F1	17	26	
F2	6	6	
F3	20	3	
F4	24	2	
ALT (U/L)*	100.2 (±10)	47 (±4.7)	<0.0001
AST (U/L)**	76.7 (±15)	25.2 (±1.4)	<0.0001
HCV-RNA (IU/mL × 10^6^)	5.8 (±2.5)	/	
HBV-DNA (IU/mL × 10^6^)	/	14.4 (±6.1)	
**HCV genotype:**			
1a	6 (8.9%)		
1b	39 (58.2%)		
2	16(23.9%)		
3	4 (6%)		
4	2 (3%)		
	—		
**Previous AVT response:**			
Naïve	35	50	
Experienced	32	/	
**DAA treatment:**			
3D°	8		
3D + RBV	8		
Sofosbuvir + Daclatasvir	4		
Sofosbuvir + Daclatasvir + RBV	5		
Sofosbuvir + Ledipasvir	7		
Sofosbuvir + Ledipasvir + RBV	7		
Sofosbuvir + Simeprevir	3		
Sofosbuvir + Simeprevir + RBV	5		
Sofosbuvir + RBV	18		
PegIFN + Telaprevir + RBV	1		
PegIFN + Sofosbuvir + RBV	1		

Data are expressed as mean ± standard error; ^Based on liver stiffness assessed by FibroScan; *Alanine Aminotransferase (ALT) normal range: 12–65 U/L; **Aspartate Aminotransferase normal range: 15–37 U/L; AVT: Antiviral Treatment; °3D: ombitasvir, paritaprevir + ritonavir, dasabuvir.

### NMR sample preparation

Following standard operating procedures, fresh serum samples were stored at −80 °C, and then thawed at room temperature at the time of analysis^57^. A total of 350 µL of a phosphate sodium buffer (70 mM Na_2_HPO_4_; 20% (v/v) ^2^H_2_O; 0.025% (v/v) NaN_3_; 0.8% (w/v) sodium trimethylsilyl [2,2,3,3-^2^H_4_]-propionate (TMSP) pH 7.4) was added to 350 µL of each serum sample, and the mixture was homogenized by vortexing for 30 s. A total of 600 µL of this mixture was transferred into a 5 mm NMR tube (Bruker BioSpin srl) for analysis.

### NMR analysis

NMR measurements were performed at the CERM/CIRMMP center for Nuclear Magnetic Resonance, University of Florence, Italy.

One-dimensional (1D) ^1^H NMR spectra for all samples were acquired using a Bruker 600 MHz spectrometer (Bruker BioSpin) operating at 600.13 MHz proton Larmor frequency and equipped with a 5 mm PATXI ^1^H-^13^C-^15^N and ^2^H-decoupling probe including a z axis gradient coil, an automatic tuning-matching (ATM) and an automatic and refrigerated sample changer (SampleJet). A BTO 2000 thermocouple served for temperature stabilization at the level of approximately 0.1 K on the sample. Before measurement, to equilibrate temperature at 310 K, samples were kept inside the NMR probe head for at least 5 minutes.

For each sample two one-dimensional proton NMR spectra were acquired with different pulse sequences, allowing the selective detection of different molecular components^58^:

(i) A standard nuclear Overhauser effect spectroscopy pulse sequence NOESY 1Dpresat (noesygppr1d.comp; Bruker BioSpin) pulse sequence, using 32 scans, 98304 data points, a spectral width of 18,029 Hz, an acquisition time of 2.7 s, a relaxation delay of 4 s and a mixing time of 0.01 s, was applied to obtain a spectrum in which both signals of metabolites and high molecular weight macromolecules are visible (lipids and lipoproteins, Supplementary Fig. 3).
(ii) A standard spin echo Carr-Purcell-Meiboom-Gill (CPMG)^59^(cpmgpr1d.comp; Bruker BioSpin) pulse sequence applied to a standard 1D sequence, with 32 scans, 73728 data points, a spectral width of 12,019 Hz and a relaxation delay of 4 s, was used for the selective observation of small molecule components present in solutions (via T2 filtering) reducing signals arising from macromolecules (Supplementary Fig. 3). The acquisition conditions used in this work are perfectly in line with the current SOPs^60^. The same conditions are also recommended by Bruker (NMR manufacturer) for metabolomic analysis^61^.

### Spectral processing and Statistical analysis

Free induction decays were multiplied by an exponential function equivalent to 0.3 Hz line-broadening factor before applying Fourier transform. Transformed spectra were automatically corrected for phase and baseline distortions and calibrated to anomeric glucose doublet at 5.24 ppm using an NMR processing software (Topspin version 3.5 pl 7 Bruker BioSpin srl). Each 1D spectrum in the range 0.2 to 10.00 ppm was segmented into 0.02 ppm chemical shift bins, and the corresponding spectral areas were integrated using AMIX software (version 3.8.4, Bruker BioSpin). Binning is a means to reduce the number of total variables and to compensate for small shifts in the signals, making the analysis more robust and reproducible^62,63^. Regions between 4.23 and 5.11 ppm containing residual water signal were removed, and the dimension of the system was reduced to 447 bins. The total spectral area was calculated on the remaining bins, and the area of each bin was normalized using PQN (Probabilistic Quotient Normalization) algorithm^64^ prior to pattern recognition. Variables were mean centered without scaling. All data analyses were performed using R, an open source software for the statistical analysis of data^65^. Multivariate data analysis was conducted on processed NMR spectra.

Principal component analysis (PCA) was used as the first exploratory analysis.

When the unsupervised approach was not effective in obtaining discrimination between groups of interest, Orthogonal Projections to Latent Structures-Discriminant Analysis (OPLS-DA), was chosen as a supervised technique. OPLS-DA is a multivariate projection method which is frequently applied for modelling spectroscopic data. This algorithm is able to separate “response-related” and “response-orthogonal” variations in data, providing benefits in terms of model interpretation compared to PCA or to PLS^66^. Multilevel partial least square (MPLS, R script developed in-house)^67^ analysis was applied to study the within-subject changes introduced in the individual metabolic profile through DAAs therapy, considering serum samples collected during HCV infection (baseline), 12 weeks (SVR12) and 24 weeks (SVR24) after the sustained virological response. All the accuracies reported and the confusion matrix for different classifications were assessed by means of 100 cycles of a Monte Carlo cross-validation scheme (MCCV, R script developed in-house). In this case, 90% of the data were randomly chosen at each iteration as a training set to build the model. Then the remaining 10% was tested, and sensitivity, specificity and accuracy of the classification were assessed.

31 metabolites were identified in the spectra by the use of matching routines of AMIX 3.8.4 (Bruker BioSpin) in combination with the BBIOREFCODE database (Bruker BioSpin), public databases (e.g. HMBD) storing reference and published literature when available.

Each metabolite was aligned to a reference value of chemical shift, obtaining a perfect alignment among all the spectra. The relative concentrations (expressed in arbitrary units) of the various metabolites were calculated by integrating the corresponding signals in defined spectral range^68^, using a home-made script for signal deconvolution and normalized using PQN algorithm, then were analyzed to determine the discriminating metabolites among the groups under study. The Wilcoxon test was chosen to infer differences between two groups of subjects^69^. The Kruskal-Wallis test followed by Dunn post-hoc analysis was chosen to infer differences among fibrosis groups (F0/F1/F2 vs F3 vs F4). The Friedman test followed by post-hoc Nemenyi analysis was chosen to ascertain pairwise differences among baseline (HCV), SVR12 and SVR24 groups. False discovery rate correction was applied using the Benjamini & Hochberg method (FDR), an adjusted *P*-value < 0.05 was considered statistically significant^70^. Changes in metabolite levels are calculated as the log_2_Fold-Change (FC) ratio of the normalized median intensities of the corresponding signals in the spectra of the two groups. The effect sizes were calculated both for pairwise comparisons (i.e. same individuals at different time points) and for groups comparisons (i.e. individuals in different groups of HBV, HCV, HC) using Cliff’s delta (Cd) formulation^71^, to aid in the identification of the meaningful signals giving an estimation of the magnitude of the separation in the different comparisons. Magnitude is assessed using the thresholds provided in Romano *et al*.^72^, i.e., |Cd| < 0.147 “negligible”, |Cd| < 0.33 “small”, |Cd| < 0.474 “medium”, otherwise “large”. MetaboAnalyst free online software was used for pathway analysis^73^.

### Ethical guidelines

The study was conducted according to the ethical guidelines of the 1975 Declaration of Helsinki; all the anti-HCV treatments were open-label and provided by the National Health Care System, therefore, ethical approval was not required for their administration. All the subjects included in the study signed an informed consent form. The local institutional review board (Comitato Etico Area Vasta Centro, AOU Careggi, Florence, Italy) approved the study (#10650_bio).

## Supplementary information

Supplementary Information
