# Supplementary material for: The metabolic fingerprints of HCV and HBV infections studied by Nuclear Magnetic Resonance Spectroscopy
Source: Sci Rep. 2019 Mar 11;9:4128. doi: 10.1038/s41598-019-40028-4 (PMC6412048; doi:10.1038/s41598-019-40028-4)
Supplement: Supplementary file 1 — Supplementary Information [file 41598_2019_40028_MOESM1_ESM.pdf]

The metabolic fingerprints of HCV and HBV infections studied by Nuclear Magnetic Resonance Spectroscopy.

Gaia Meoni[1,2+], Serena Lorini[3,+], Monica Monti[3], Francesco Madia[3], Giampaolo Corti[4], Claudio Luchinat[1,2,5], Anna Linda Zignego[3], Leonardo Tenori[1,2,6,*] and Laura Gragnani[3,*]

[1]University of Florence, CERM, Sesto Fiorentino, 50019, Italy
[2]C.I.R.M.M.P., Sesto Fiorentino, 50019, Italy
[3]Careggi University Hospital, Department of Experimental and Clinical Medicine, Interdepartmental Center for Systemic Manifestations of Hepatitis Viruses (MaSVE), Florence, 50134, Italy
[4]Careggi University Hospital, Infectious and Tropical Diseases Unit, Florence, 50134, Italy
[5]University of Florence, Department of Chemistry "Ugo Schiff", Sesto Fiorentino, 50019, Italy
[6]University of Florence, Department of Experimental and Clinical Medicine, Florence, 50134, Italy

Supplementary Figure 1

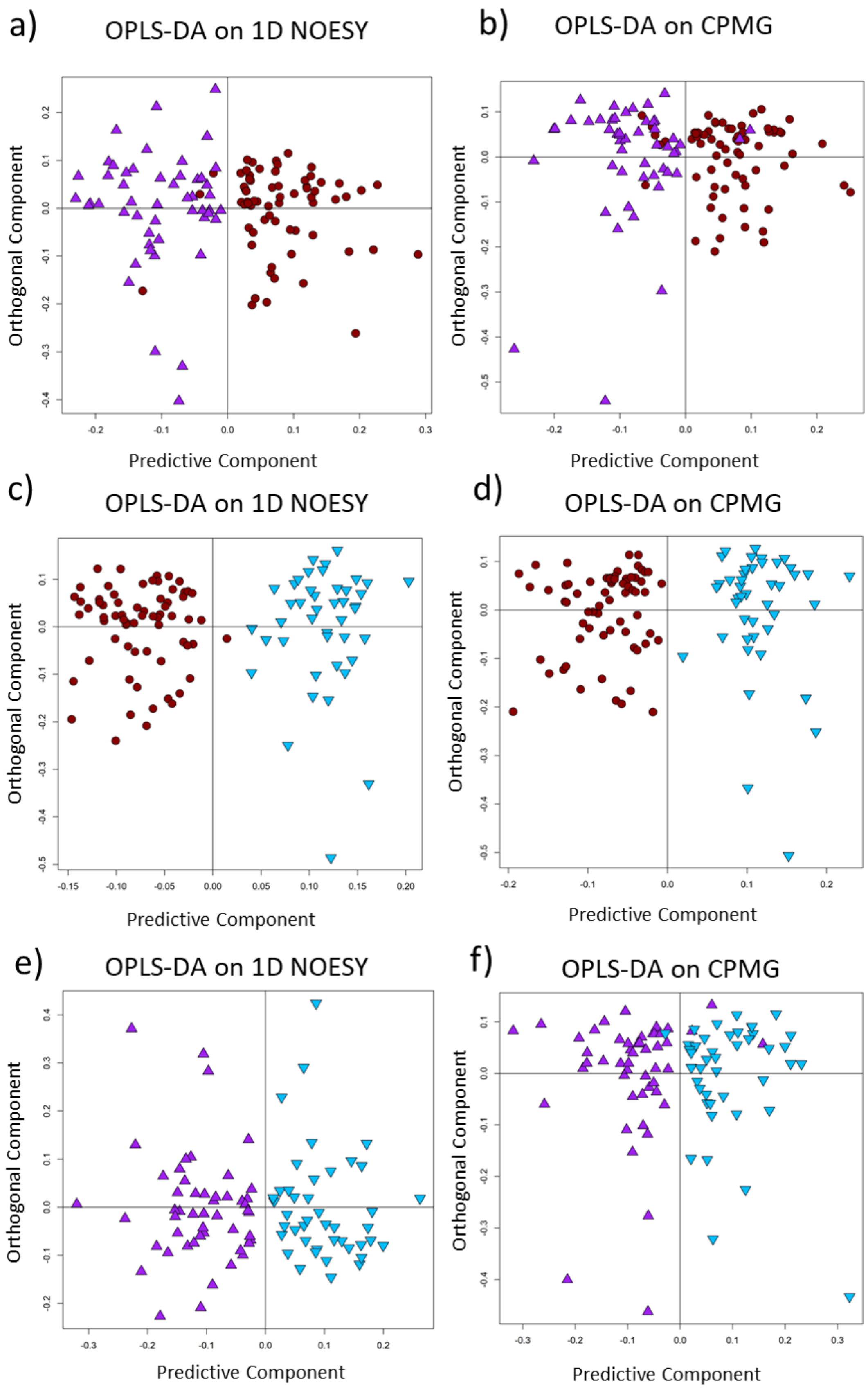

Score plots of OPLS-DA, discriminating HCV (red dots, n = 67), HBV patients (purple triangles, n = 50) and HC (sky-blue triangles, n =43) using both experiments (1D NOESY and GPMG). a,b) HCV vs HBV; c,d) HCV vs HC; e,f) HBV vs HC.

Supplementary Table 1. Confusion matrix and predictive accuracy of Monte Carlo cross-validation are reported for both 1D NOESY and CPMG models.

	NOESY			CPMG	
	Confusion matrix			*Confusion matrix*	
	**HCV**	**HBV**		**HCV**	**HBV**
**HCV**	85.7	14.3	**HCV**	81.3	18.7
**HBV**	13.6	86.3	**HBV**	12	88
Predictive accuracy:	**86%**		*Predictive accuracy*:	**84%**	
	Confusion matrix			*Confusion matrix*	
	**HCV**	**HC**		**HCV**	**HC**
**HCV**	97.9	2.1	**HCV**	96.8	3.2
**HC**	0	100	**HC**	0.2	99.8
Predictive accuracy:	**98.7%**		*Predictive accuracy:*	**97.9%**	
	Confusion matrix			*Confusion matrix*	
	**HBV**	**HC**		**HBV**	**HC**
**HBV**	73.1	26.9	**HBV**	72.4	27.6
**HC**	11.6	88.4	**HC**	16.3	83.7
Predictive accuracy:	**80.1%**		*Predictive accuracy:*	**77.6%**	

Supplementary Table 2. Concentrations in arbitrary units [log(mean ± SD)] of the metabolites assigned (MSI level 1) in HCV, HBV and HC serum samples. Significantly different *P*-values from each comparison are reported in bold. The magnitude of the effect size is assessed using the thresholds provided in (Romano 2006) and is reported as "n" as "negligible", "s" as "small", "m" as "medium" and "l" as "large".

	HCV (67 subjects)			HBV (50 subjects)			HC (43 subjects)			HCV vs HC	HCV vs HBV	HBV vs HC
Metabolites	***mean***	**±**	***SD***	***mean***	**±**	***SD***	***mean***	**±**	***SD***	***P-value (effect size)***	***P-value (effect size)***	***P-value (effect size)***
valine	9.36E+02	±	1.81E+02	1.00E+03	±	2.11E+02	9.80E+02	±	2.14E+02	4.81E-01(n)	1.46E-01(s)	6.21E-01(n)
isoleucine	1.17E+02	±	3.35E+01	1.45E+02	±	4.74E+01	1.32E+02	±	3.77E+01	**4.46E-02(s)**	**8.13E-03(m)**	4.98E-01(n)
leucine	2.23E+02	±	6.96E+01	2.70E+02	±	9.68E+01	2.31E+02	±	8.14E+01	8.58E-01(n)	**1.61E-02(s)**	9.07E-02(s)
isobutyrate	3.34E+01	±	1.10E+01	3.02E+01	±	9.99E+00	2.96E+01	±	7.55E+00	1.55E-01(s)	1.95E-01(s)	9.42E-01(n)
2-methylsuccinate	1.42E+01	±	1.22E+01	1.39E+01	±	1.43E+01	1.21E+01	±	1.20E+01	3.70E-01(n)	5.86E-01(s)	8.76E-01(n)
3-hydroxybutyrate	3.45E+01	±	3.22E+01	2.09E+01	±	2.15E+01	1.58E+01	±	2.12E+01	**2.39E-05(l)**	**2.24E-02(s)**	**3.08E-02(s)**
lactate	4.19E+03	±	1.20E+03	3.62E+03	±	1.77E+03	2.42E+03	±	8.00E+02	**3.62E-11(l)**	**3.06E-02(s)**	**2.76E-03(m)**
alanine	1.41E+03	±	3.15E+02	1.47E+03	±	4.44E+02	1.39E+03	±	3.49E+02	9.71E-01(n)	8.21E-01(n)	8.74E-01(n)
acetate	1.88E+02	±	1.75E+02	3.78E+02	±	3.89E+02	6.34E+01	±	3.11E+01	**2.41E-10(l)**	3.58E-01(n)	**2.20E-06(l)**
acetone	2.77E+02	±	2.13E+02	2.16E+02	±	1.36E+02	1.92E+02	±	1.37E+02	**1.67E-03(m)**	1.19E-01(s)	3.97E-01(s)
glutamate	1.95E+02	±	8.42E+01	2.40E+02	±	1.43E+02	1.36E+02	±	5.90E+01	**6.54E-05(l)**	5.20E-01(n)	**2.05E-03(m)**
pyruvate	3.24E+02	±	1.54E+02	2.13E+02	±	1.50E+02	1.86E+02	±	8.00E+01	**7.39E-07(l)**	**1.97E-05(l)**	8.76E-01(n)

glutamine	1.30E+02	±	4.77E+01	9.75E+01	±	6.90E+01	1.70E+02	±	3.80E+01	**6.22E-05(l)**	7.53E-02(s)	**4.64E-06(l)**
citrate	9.42E+01	±	3.50E+01	8.17E+01	±	3.46E+01	9.26E+01	±	2.66E+01	6.35E-01(n)	1.51E-01(s)	6.21E-01(n)
phenylalanine	2.44E+02	±	5.72E+01	2.21E+02	±	5.77E+01	2.14E+02	±	5.14E+01	**2.23E-03(m)**	**1.40E-02(m)**	7.33E-01(n)
dimethylamine	3.29E+01	±	2.17E+01	2.19E+01	±	1.57E+01	5.20E+01	±	7.45E+01	8.00E-01(n)	**2.08E-02(s)**	**1.27E-02(m)**
choline	3.25E+02	±	2.37E+02	5.49E+02	±	7.08E+02	4.63E+01	±	1.47E+02	**1.39E-11(l)**	8.52E-01(n)	**2.52E-07(l)**
sarcosine	4.82E+01	±	1.25E+02	8.09E+01	±	9.54E+01	7.41E+01	±	1.65E+02	9.21E-01(n)	5.07E-02(s)	7.25E-02(s)
2-oxoglutarate	2.94E+01	±	3.11E+01	1.88E+01	±	4.30E+01	6.81E+00	±	5.10E+00	**1.52E-09(l)**	**1.38E-05(l)**	9.42E-01(n)
ornithine	6.86E+01	±	3.75E+01	6.33E+01	±	4.76E+01	3.28E+01	±	1.70E+01	**1.27E-08(l)**	1.34E-01(s)	**5.44E-03(m)**
proline	5.09E+01	±	1.45E+01	5.91E+01	±	2.03E+01	5.31E+01	±	2.11E+01	9.71E-01(n)	8.28E-02(s)	2.52E-01(s)
glycine	5.84E+02	±	1.51E+02	6.28E+02	±	1.35E+02	6.25E+02	±	1.19E+02	9.85E-02(s)	9.16E-02(s)	9.33E-01(n)
glycerol	2.30E+02	±	8.21E+01	2.42E+02	±	9.95E+01	2.34E+02	±	1.44E+02	8.71E-01(n)	5.82E-01(n)	6.21E-01(n)
creatine	8.23E+01	±	5.12E+01	1.12E+02	±	7.44E+01	1.01E+02	±	6.70E+01	2.32E-01(s)	9.16E-02(s)	7.07E-01(n)
tyrosine	1.67E+02	±	5.57E+01	1.43E+02	±	4.05E+01	1.30E+02	±	3.72E+01	**8.23E-04(m)**	8.28E-02(s)	2.72E-01(s)
creatinine	1.72E+02	±	5.20E+01	2.31E+02	±	2.75E+02	1.90E+02	±	4.88E+01	1.15E-01(s)	8.28E-02(s)	9.42E-01(n)
glucose	2.43E+03	±	1.14E+03	2.42E+03	±	1.07E+03	2.86E+03	±	4.81E+02	**1.22E-05(l)**	5.86E-01(n)	**2.55E-04(l)**
histidine	1.25E+02	±	2.31E+01	1.51E+02	±	1.43E+02	1.29E+02	±	2.63E+01	4.01E-01(n)	2.93E-01(n)	8.64E-01(n)
formate	1.78E+01	±	7.20E+00	2.15E+01	±	1.28E+01	8.91E+00	±	2.87E+00	**2.11E-12(l)**	5.86E-01(n)	**2.28E-07(l)**
fumarate	6.34E+00	±	2.87E+00	4.07E+00	±	2.55E+00	1.73E+00	±	1.08E+00	**3.64E-13(l)**	**1.66E-04(m)**	**2.99E-06(l)**
dimethyl sulfone	1.07E+02	±	2.51E+02	4.91E+01	±	4.14E+01	5.55E+01	±	2.37E+01	9.71E-01(n)	5.07E-02(s)	**7.25E-02(s)**

Supplementary Figure 2

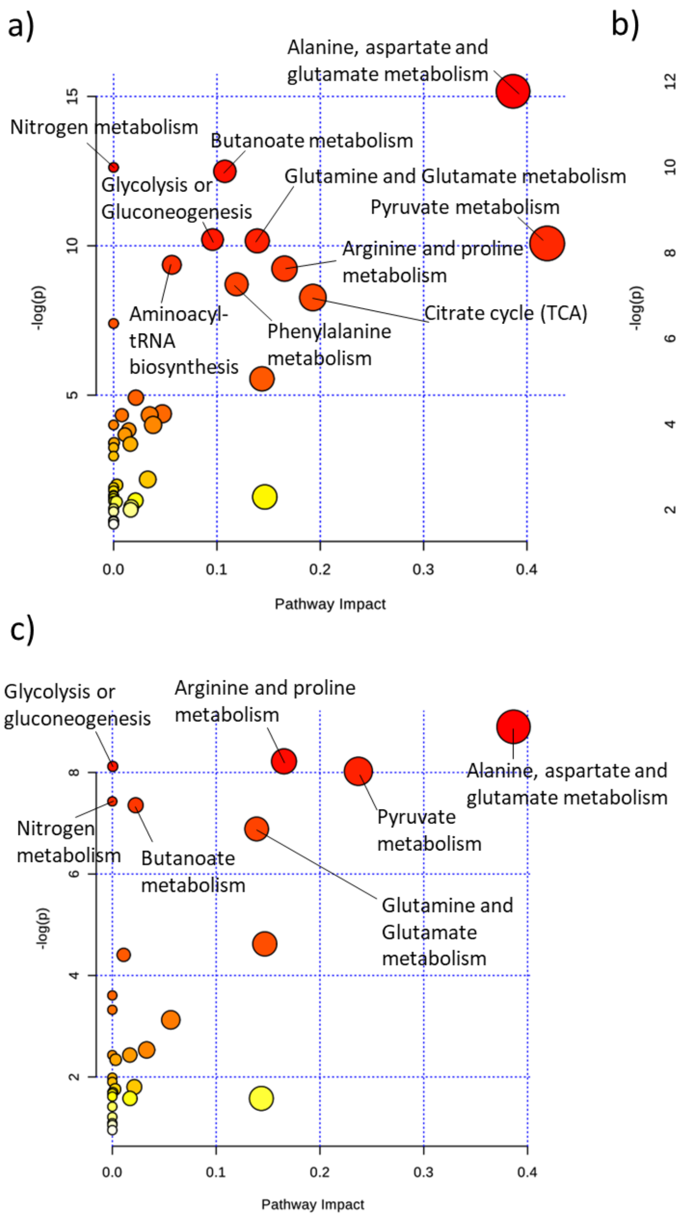

Summary of pathway analysis of significantly altered serum metabolites, carried out using MetaboAnalyst software 4.0.a) most contributing metabolic pathways considering HCV and HC serum metabolites; b) most contributing metabolic pathways considering HCV and HBV serum metabolites; C) most contributing metabolic pathways considering HBV and HC serum metabolites. Detailed data are reported in supplementary table 3.

Supplementary Table 3. An integrated analysis based on MetaboAnalyst 4.0 software built on significantly altered metabolites in patients (HCV and HBV positive) and controls (HC): view of most contributing pathways; *P*-value is the original *P*-value calculated from the enrichment analysis; Holm *P*-value is the *P*-value adjusted by Holm-Bonferroni method; the FDR is the *P*-value adjusted using False Discovery Rate; the Impact is the pathway impact value calculated from pathway topology analysis.

**HCV-HC**					
**Pathway Name**	**Metabolites**	***P*-value**	**Holm P-value**	**FDR**	**Impact**
Alanine, aspartate and glutamate metabolism	Pyruvate; 2-oxoglutarate; glutamine; glutamate; fumarate	$2.6x10^{-7}$	$2x10^{-5}$	$2x10^{-5}$	0.39
Nitrogen metabolism	Phenylalanine; tyrosine; glutamate; glutamine; formate	$3.3x10^{-6}$	$2.6x10^{-4}$	$9.9X10^{-5}$	0
Butanoate metabolism	3-hydroxybutyrate; glutamate; pyruvate; fumarate; 2-oxoglutarate	$3.7x10^{-6}$	$2.9x10^{-4}$	$9.9x10^{-5}$	0.12
Glycolysis or Gluconeogenesis	Pyruvate; lactate; glucose; acetate	$3.7x10^{-5}$	0.003	$5.6x10^{-4}$	0.09
D-Glutamine and D-glutamate metabolism	Glutamate; glutamine; 2-oxoglutarate	3.810^{-5}	0.003	$5.6x10^{-4}$	0.14
Pyruvate metabolism	Pyruvate; lactate; formate; acetate	$4.2x10^{-5}$	0.003	$5.6x10^{-4}$	0.42
Aminoacyl-tRNA biosynthesis	Phenylalanine; glutamine; isoleucine; tyrosine; glutamate	$8.6x10^{-5}$	0.006	$9.7x10^{-4}$	0.05
Arginine and proline metabolism	Glutamine; ornithine; glutamate; fumarate; pyruvate	$9.7x10^{-5}$	0.007	$9.7x10^{-4}$	0.16
Phenylalanine metabolism	Phenylalanine; pyruvate; fumarate; tyrosine	$1.6x10^{-4}$	0.012	0.001	0.12
Citrate cycle (TCA cycle)	2-oxoglutarate; pyruvate; fumarate	$2.6x10^{-4}$	0.02	0.002	0.19
Synthesis and degradation of ketone bodies	3-hydroxybutyrate; acetone	$6.1x10^{-4}$	0.04	0.004	0
Glyoxylate and dicarboxylate metabolism	2-oxoglutarate; formate; pyruvate	0.0034	0.27	0.027	0.14
Taurine and hypotaurine metabolism	Pyruvate; acetate	0.007	0.5	0.04	0.02
**HCV-HBV**					
Butanoate metabolism	3-hydroxybutyrate; pyruvate; 2-oxoglutarate; fumarate	$7.7x10^{-6}$	$6.2x10^{-4}$	$6.2x10^{-4}$	0.12
Citrate cycle (TCA cycle)	2-oxoglutarate; pyruvate; fumarate	$3.99x10^{-5}$	0.003	0.0015	0.19
Alanine, aspartate and glutamate metabolism	Pyruvate; 2-oxoglutarate; fumarate	$7.04x10^{-5}$	0.005	0.0018	0.003
Valine, leucine and isoleucine biosynthesis	Pyruvate; leucine; isoleucine	$1.1x10^{-4}$	0.0077	0.002	0.05

Phenylalanine metabolism	Phenylalanine; pyruvate; fumarate	$4.74x10^{-4}$	0.036	0.0076	0.12
Aminoacyl-tRNA biosynthesis	Phenylalanine; isoleucine; leucine	0.002	0.1	0.02	0
Glycolysis or Gluconeogenesis	Pyruvate; lactate	0.006	0.4	0.05	0.1
Vitamin B6 metabolism	2-oxoglutarate; pyruvate	0.006	0.4	0.05	0.04
Pyruvate metabolism	Pyruvate; lactate	0.006	0.4	0.05	0.3
**HBV-HC**					
Alanine, aspartate and glutamate metabolism	Glutamine; glutamate; fumarate	$1.4x10^{-4}$	0.01	0.006	0.4
Arginine and proline metabolism	Glutamine; ornithine; glutamate; fumarate	$2.7x10^{-4}$	0.02	0.006	0.16
Glycolysis or Gluconeogenesis	Lactate; glucose; acetate	$2.9x10^{-4}$	0.02	0.006	$4.6x10^{-4}$
Pyruvate metabolism	Lactate; formate; acetate	$3.3x10^{-4}$	0.02	0.006	0.24
Nitrogen metabolism	Glutamate; glutamine; formate	$5.9x10^{-4}$	0.04	0.008	0
Butanoate metabolism	3-hydroxybutyrate; glutamate; fumaric acid	$6.4x10^{-4}$	0.05	0.008	0.02
Glutamine and glutamate metabolism	Glutamate; glutamine	0.001	0.07	0.01	0.14

Supplementary Figure 3.

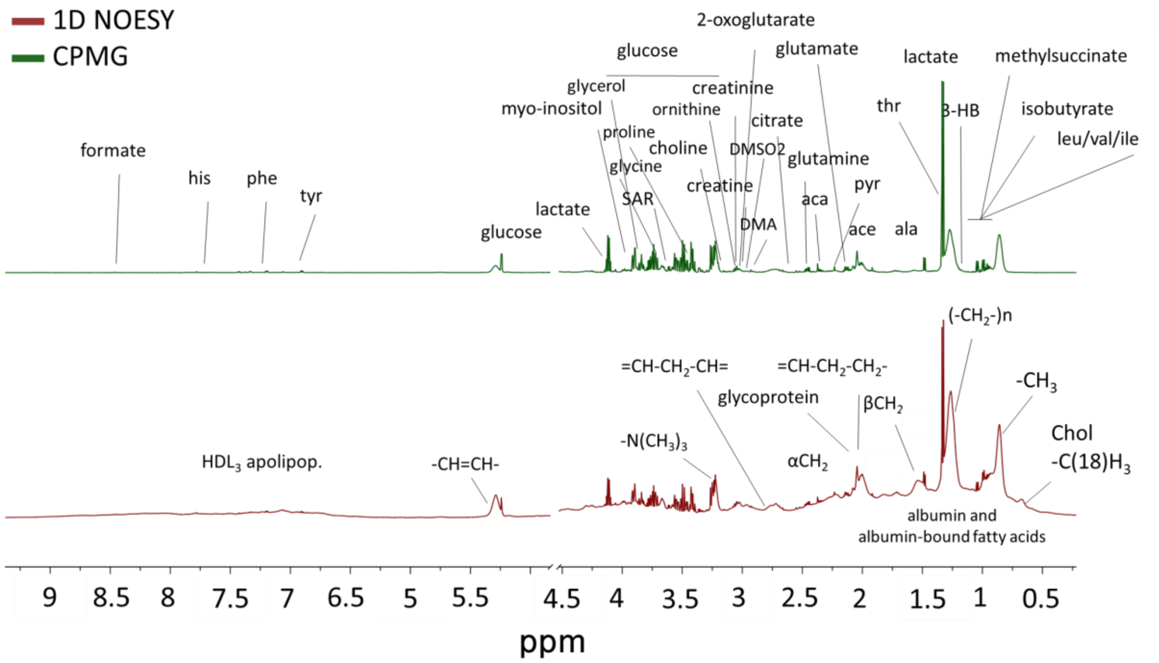

From the bottom to the top: 1D NOESY (red one) and CPMG (green) serum spectra of a HCV positive patient. Some of the main peaks are assigned. Serum lipoprotein lipids and albumin are the main ones responsible for the broad signals. The residual water peak region (4.5-5 ppm) is not shown.

Supplementary Figure 4.

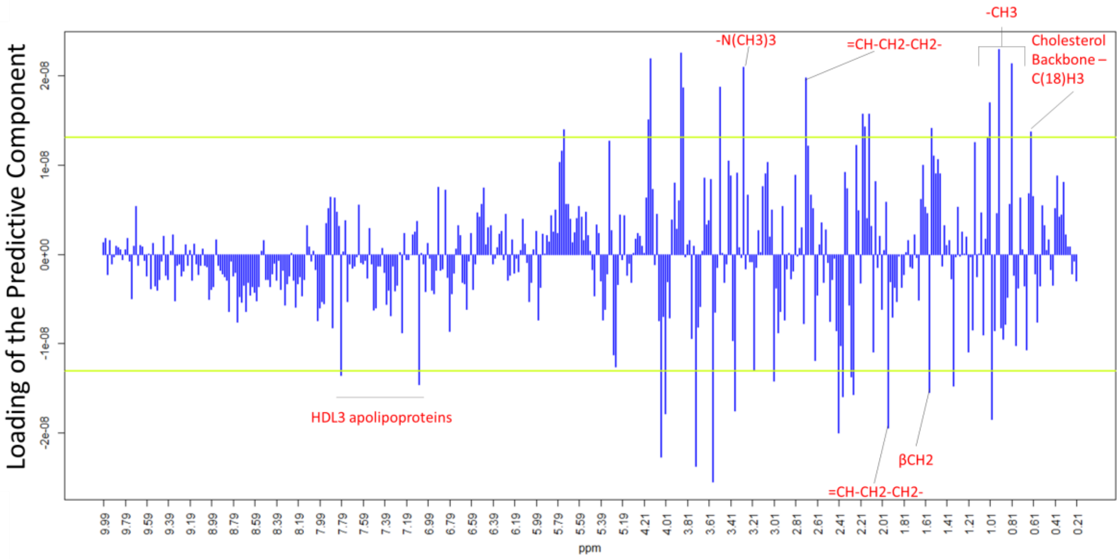

Loadings bar-plots of 1D NOESY OPLS-DA model. Discrimination among different viral infections (see supplementary figure 1a), showing that part of the most contributing variable to the separation of HCV from HBV infected patients can be attributed to NMR signals arising from macromolecule. Positive loading values are attributed to HCV infection; while negative values of the loading are attributed to HBV viral infection. Yellow line represents the mean value +/- two times standard deviation**.**

Supplementary Table 4. Concentrations in arbitrary units (mean ± SD) of the metabolites assigned (MSI level 1) in HCV infected patients (Baseline) with different fibrosis levels. Significantly different *P*-values from the comparisons are also reported. The magnitude of the effect size is assessed using the thresholds provided in (Romano 2006) and is reported as "n" as "negligible", "s" as "small", "m" as "medium" and "l" as "large".

	F0/F1/F2 (23 subjects)			F3 (20 subjects)			F4 (24 subjects)			
METABOLITES	***mean***	***±***	***sd***	***mean***	***±***	***sd***	***mean***	***±***	***sd***	***P-values***
VALINE	872.9914	±	143.0722	923.4722	±	187.1931	1006.024	±	190.0334	F0/F2 vs F4 = 0.035(m)
ISOLEUCINE	110.2645	±	19.31842	111.8576	±	22.85543	128.9745	±	47.12133	
LEUCINE	200.7073	±	44.30786	245.6707	±	99.42027	226.0607	±	53.89349	
ISOBUTYRATE	28.92624	±	9.42736	31.1681	±	9.391529	39.54155	±	11.32954	F0/F2 vs F4 = 0.003(l) F3 vs F4 = 0.027(s)
2-METHYLSUCCINATE	12.88537	±	11.60661	14.58815	±	12.79503	15.01337	±	12.72507	
3-HYDROXYBUTYRATE	34.59433	±	39.54477	27.37601	±	16.23305	40.35884	±	34.34469	
LACTATE	4192.145	±	1330.22	4040.164	±	1028.309	4323.169	±	1244.042	
ALANINE	1357.024	±	321.4859	1391.873	±	357.5795	1462.398	±	274.0975	
ACETATE	195.8567	±	204.7201	178.3808	±	162.089	188.6576	±	160.5097	
ACETONE	284.5237	±	204.513	247.2592	±	113.2241	293.6201	±	281.1121	
GLUTAMATE	190.328	±	80.25048	179.9669	±	85.29092	211.5896	±	87.43645	
PYRUVATE	327.7061	±	126.1987	295.7834	±	118.7823	343.4813	±	200.7553	
GLUTAMINE	134.1233	±	43.94446	129.7077	±	51.69462	126.7755	±	49.41918	
CITRATE	89.5828	±	33.52876	92.89142	±	26.01442	99.79614	±	42.63248	
PHENYLALANINE	227.8844	±	35.16247	241.4996	±	45.19537	261.9931	±	76.80927	F0/F2 vs F4 = 0.016(m)
DIMETHYLAMINE	30.78331	±	18.88965	33.22431	±	24.29584	34.73956	±	22.79983	
CHOLINE	310.8827	±	206.8082	356.9217	±	332.3066	312.6665	±	167.7154	
SARCOSINE	27.86382	±	53.41223	29.34722	±	28.45348	83.39211	±	198.9539	
2-OXOGLUTARATE	20.62694	±	28.94839	31.5553	±	31.95039	36.05691	±	31.67711	F0/F2 vs F4 = 0.009(m)
ORNITHINE	61.21947	±	31.49703	58.22912	±	32.29505	84.20022	±	42.73492	
PROLINE	49.64702	±	15.25496	46.73222	±	12.31658	55.66286	±	14.62757	
GLYCINE	652.331	±	201.0095	556.2076	±	110.009	542.8855	±	97.90495	
GLYCEROL	200.0959	±	59.66613	256.951	±	112.5743	235.3082	±	62.6684	
CREATINE	95.98974	±	43.17941	70.11794	±	48.1848	79.28722	±	59.09022	
TYROSINE	135.6254	±	30.91254	167.2505	±	58.11155	196.0381	±	57.72867	F0/F2 vs F3 = 0.042(m)<br>F0/F2 vs F4 = 0.0001(l)
CREATININE	174.6617	±	62.66421	180.5294	±	43.83221	161.2994	±	47.20629	
GLUCOSE	2365.527	±	1124.358	2471.909	±	1177.919	2448.565	±	1170.817	
HISTIDINE	125.9417	±	24.67217	119.8494	±	18.24007	127.6986	±	25.24045	
FORMATE	14.03734	±	4.017313	19.93502	±	9.969093	19.60409	±	5.468339	F0/F2 vs F3 = 0.013 (m)<br>F0/F2 vs F4 = 0.001(l)
FUMARATE	5.505981	±	2.52722	6.333263	±	3.267491	7.135679	±	2.701265	
DIMETHYL SULFONE	156.0876	±	354.3236	113.2424	±	256.8157	54.24472	±	30.75187	

Supplementary Table 5. Concentrations in arbitrary units (mean ± SD) of the metabolites assigned (MSI level 1) in SVR12 patients with different fibrosis levels. Significantly different *P*-values from the comparisons are also reported. The magnitude of the effect size is assessed using the thresholds provided in (Romano 2006) and is reported as “n” as “negligible”, “s” as “small”, “m” as “medium” and “l” as “large”.

	F0/F1/F2 (23 subjects)			F3 (20 subjects)			F4 (24 subjects)			
Metabolites	***mean***	***±***	***sd***	***mean***	***±***	***sd***	***mean***	***±***	***sd***	***P-values***
VALINE	878.5802	±	164.8128	978.1091	±	183.3603	982.1636	±	220.9057	
ISOLEUCINE	104.983	±	30.7078	119.1752	±	26.12661	119.0149	±	35.30863	
LEUCINE	206.8827	±	54.06354	229.4831	±	62.25033	229.3056	±	84.19148	
ISOBUTYRATE	28.47156	±	8.655182	33.3635	±	9.604465	38.32618	±	15.94306	
2-METHYLSUCCINATE	14.16253	±	10.15293	15.3847	±	13.15978	15.65041	±	16.00054	
3-HYDROXYBUTYRATE	17.18685	±	14.59583	36.56811	±	38.34298	19.81219	±	14.34052	
LACTATE	3548.522	±	1168.12	4358.056	±	1657.846	4272.276	±	1124.701	
ALANINE	1330.031	±	266.4937	1401.785	±	395.6161	1487.159	±	304.2056	
ACETATE	90.60917	±	39.42667	122.1464	±	93.28265	118.9622	±	107.4117	
ACETONE	205.4372	±	91.46179	236.2751	±	121.9978	266.6749	±	186.8173	
GLUTAMATE	160.6675	±	47.72061	179.4132	±	76.16655	198.8988	±	79.22456	
PYRUVATE	335.9581	±	109.8447	329.1377	±	200.3567	352.4094	±	116.4868	
GLUTAMINE	148.8389	±	25.55513	136.4283	±	35.71063	125.9396	±	35.889	F0/F2 vs F4 = 0.045 (m)
CITRATE	102.4793	±	27.17361	82.59975	±	39.92617	93.14139	±	40.03672	
PHENYLALANINE	221.0171	±	54.35739	230.2129	±	71.01162	265.3839	±	69.20688	F0/F2 vs F4 = 0.043 (m)
DIMETHYLAMINE	43.25279	±	58.58497	31.89837	±	23.68526	39.50236	±	27.82123	
CHOLINE	213.7519	±	139.3133	270.4795	±	163.4079	293.3026	±	189.2334	
SARCOSINE	18.77254	±	22.27758	58.04898	±	90.9906	104.0718	±	232.2182	
2-OXOGLUTARATE	14.43368	±	25.49835	21.22785	±	32.74073	16.33092	±	7.656204	F0/F2 vs F4 = 0.01 (m)
ORNITHINE	50.46928	±	23.95702	61.22086	±	34.84361	71.62092	±	41.2111	
PROLINE	50.40539	±	16.80852	55.72947	±	16.19476	60.80946	±	29.305	
GLYCINE	669.1007	±	113.7242	606.4909	±	119.1829	592.9336	±	145.5215	
GLYCEROL	246.909	±	207.7596	212.0495	±	58.90802	219.0967	±	77.10528	
CREATINE	90.11979	±	55.56252	68.48192	±	35.92777	83.00246	±	71.26684	
TYROSINE	129.664	±	31.5404	153.1314	±	42.15222	184.9529	±	83.53053	F0/F2 vs F4 = 0.043 (m)
CREATININE	175.5075	±	67.02307	186.1984	±	34.17521	161.9486	±	62.40044	
GLUCOSE	2501.656	±	1233.932	2364.464	±	767.7128	2509.339	±	956.2962	
HISTIDINE	120.0146	±	25.81705	128.8307	±	22.84923	132.3853	±	28.21402	
FORMATE	12.04921	±	5.299339	15.41144	±	7.52849	16.50948	±	9.271967	
FUMARATE	4.034736	±	2.524734	5.888089	±	3.240363	6.007497	±	2.949352	
DIMETHYL SULFONE	170.1642	±	576.6509	62.61559	±	31.3587	59.33534	±	63.91941	

Supplementary Table 6. Concentrations in arbitrary units (mean ± SD) of the metabolites assigned (MSI level 1) in SVR24 patients with different fibrosis levels. Significantly different P-values from the comparisons are also reported. The magnitude of the effect size is assessed using the thresholds provided in (Romano 2006) and is reported as “n” as “negligible”, “s” as “small”, “m” as “medium” and “l” as “large”.

	F0/F1/F2 (23 SUBJECTS)			F3 (20 SUBJECTS)			F4 (24 SUBJECTS)			
Metabolites	***mean***	**±**	***sd***	***mean***	**±**	***sd***	***mean***	**±**	***sd***	***P-values***
VALINE	816.1322	±	175.2088	910.9055	±	113.7559	864.3518	±	241.7014	
ISOLEUCINE	108.3865	±	38.68138	114.5766	±	17.27441	110.8553	±	34.13063	
LEUCINE	206.6237	±	76.45991	236.5747	±	55.28458	228.6679	±	87.43291	
ISOBUTYRATE	30.6914	±	14.60333	28.55075	±	7.728365	36.54976	±	16.60386	
2-METHYLSUCCINATE	13.50245	±	14.1309	12.4181	±	8.682088	12.16944	±	10.9797	
3-HYDROXYBUTYRATE	21.6248	±	23.30183	14.29376	±	10.53473	37.81965	±	36.98521	
LACTATE	3625.53	±	1503.181	4023.024	±	1092.116	3518.213	±	1718.244	
ALANINE	1178.56	±	274.5454	1297.342	±	175.3452	1284.896	±	357.863	
ACETATE	132.4665	±	101.229	168.8207	±	126.7609	123.8931	±	78.25261	
ACETONE	259.8753	±	149.3608	253.2759	±	110.8628	318.1729	±	239.6095	
GLUTAMATE	140.4988	±	53.49719	192.9817	±	72.45277	156.2723	±	104.9528	
PYRUVATE	305.4263	±	101.7993	306.5109	±	100.7126	299.2185	±	109.2571	
GLUTAMINE	130.4382	±	21.46937	115.3625	±	31.22951	111.3299	±	39.5484	F0/F2 vs F4 = 0.01(s)
CITRATE	86.40278	±	32.22326	68.43276	±	40.20644	93.34926	±	49.00735	
METHIONINE	41.38683	±	15.50586	49.07082	±	16.18497	41.33306	±	15.8318	
PHENYLALANINE	213.1676	±	57.20165	231.2008	±	35.69953	222.1332	±	74.65876	
DIMETHYLAMINE	49.7225	±	70.77825	40.8382	±	57.63194	46.49572	±	63.99615	
CHOLINE	239.8566	±	165.2892	270.2267	±	165.1887	153.1702	±	184.8209	
SARCOSINE	74.05955	±	179.241	85.23142	±	180.6324	171.3496	±	358.8475	
2-OXOGLUTARATE	9.918583	±	6.988234	8.224293	±	6.104633	9.971286	±	7.877852	
ORNITHINE	74.49051	±	46.1415	62.27972	±	35.83145	51.99081	±	31.99896	
PROLINE	44.28869	±	15.5671	49.98553	±	26.20892	51.12165	±	26.02578	
GLYCINE	615.0294	±	132.5945	573.5426	±	125.7025	541.423	±	146.1363	
GLYCEROL	216.0578	±	60.73088	221.7939	±	101.082	240.3614	±	154.2644	
CREATINE	59.85857	±	37.7463	75.79023	±	66.30669	52.31979	±	44.55576	
TYROSINE	108.3306	±	34.32403	120.6797	±	32.32322	153.2354	±	78.51438	
CREATININE	156.187	±	42.29091	174.3305	±	38.83627	164.1034	±	73.35583	
GLUCOSE	2014.378	±	486.6631	2087.699	±	389.9089	2610.645	±	1368.026	
HISTIDINE	107.9622	±	26.79335	115.4834	±	21.79021	106.4334	±	19.4607	
FORMATE	15.38128	±	10.70641	17.47975	±	10.60822	14.85762	±	8.666453	
FUMARATE	4.576927	±	2.806694	5.766869	±	2.588213	5.637881	±	3.11872	
DIMETHYL SULFONE	46.17509	±	23.75042	160.1803	±	445.0755	51.60176	±	23.30473	
